# Obesity is not associated with adverse outcomes among hospitalized patients with *Clostridioides difficile* infection

**DOI:** 10.1186/s13099-022-00479-z

**Published:** 2022-01-29

**Authors:** Alyyah Malick, Ying Wang, Jordan Axelrad, Hojjat Salmasian, Daniel Freedberg

**Affiliations:** 1 Division of Digestive and Liver Diseases, Columbia University Irving Medical Center, New York, USA; 2 Division of Gastroenterology and Hepatology, Robert Wood Johnson University Hospital, New Brunswick, USA; 3 Inflammatory Bowel Disease Center, Division of Gastroenterology, New York Grossman School of Medicine, New York, USA; 4 Division of General Internal Medicine, Brigham and Women’s Hospital, Boston, USA

**Keywords:** *Clostridioides difficile*, Obesity, Body mass index, Age, Mortality

## Abstract

**Background:**

Obesity is associated with increased risk for death in most infections but has not been studied as a risk factor for mortality in *Clostridioides difficile* infection (CDI). This study tested obesity as a risk factor for death in patients hospitalized with CDI. This was a three-center retrospective study that included hospitalized adults with CDI at Columbia University Irving Medical Center, Brigham and Women’s Hospital, and NYU Langone from 2010 to 2018. Multivariate logistic regression was used to assess the relationship between obesity, measured by body mass index, and death from any cause within 30 days after the index CDI test.

**Results:**

Data for 3851 patients were analyzed, including 373 (9.7%) who died within 30 days following a diagnosis of CDI. After adjusting for other factors, BMI was not associated with increased risk for death in any BMI category [adjusted OR (aOR) 0.96, 95% CI 0.69 to 1.34 for BMI > 30 vs BMI 20–30; aOR 1.02, 95% CI 0.53 to 1.87 for BMI > 40 vs BMI 20–30]. After stratifying into three groups by age, there were trends towards increased mortality with obesity in the middle-aged (56–75 vs ≤ 55 years old) yet decreased mortality with obesity in the old (> 75 vs ≤ 55) (p = NS for all). Advanced age and low albumin were the factors most strongly associated with death.

**Conclusions:**

We found no association between obesity and death among patients with CDI, in contrast to most other infections. Obesity is not likely to be useful for risk-stratifying hospitalized patients with CDI.

**Supplementary Information:**

The online version contains supplementary material available at 10.1186/s13099-022-00479-z.

## Background

*Clostridioides difficile* infection (CDI) is the most common cause of nosocomial diarrhea in the developed world and has been increasing in incidence and severity over the last two decades. There are 500,000 cases of CDI annually in the United States, causing $4.8 billion in health care costs and 29,000 deaths [1, 2]. Several potential reasons underlie the increased incidence of CDI including the rise in the North American Pulsed Field-1 (NAP-1) strain, increased use of broad-spectrum antibiotics, and altered patient demographics (i.e., older patients who have more comorbidities and therefore increased risk for CDI) [3–7].

Another potential reason for the rise in CDI may be the concurrent worldwide rise in obesity. Obesity rates have nearly tripled since 1975, and more than 650 million adults are obese, posing an increasingly significant challenge to health [8]. Obesity has been associated with death and other adverse outcomes in most infections that, like CDI, frequently require hospitalization or are associated with hospitalization. For example, in H1N1 influenza infections, obesity increases the risk of intensive care unit (ICU) admission, and morbid obesity is associated with higher risk of death [9–11]. More recently, obesity has been associated with mortality in SARS-CoV-2 infection in an age-dependent fashion [10].

Obesity causes a pro-inflammatory state that may account for its association with adverse outcomes in infections. Adipose tissue directly drives the production of cytokines such as leptin, which induces chemokines, increases neutrophil chemotaxis, enhances natural killer cell cytotoxicity, and activates dendritic cells [12]. Leptin mediates the inflammatory response to *C. difficile* toxin A, and the increase of leptin in obesity thus may partially explain the increased severity of CDI [13–15].

Obesity has not previously been studied as a risk factor for complications and mortality in CDI. Established risk factors for adverse outcomes in CDI include leukocytosis, elevated creatinine, older age and low serum albumin [16–18]. It is unknown whether these variables are optimal for prediction of CDI-associated mortality, or whether addition of obesity to these other factors may better help to predict the patients with CDI at the highest risk for death. Improved risk-stratification of hospitalized patients with CDI could focus resources on the patients with the highest likelihood of death and also help to define appropriate entry criteria for trials so that novel therapies for CDI could be tested on the patients most likely to experience adverse outcomes.

We performed a retrospective cohort study to determine whether obesity (as measured by BMI) is associated with mortality in CDI patients. We hypothesized that obesity would demonstrate a non-linear relationship with mortality, in which underweight and obese patients would have higher mortality compared to normal weight individuals. We further hypothesized that there would be effect modification of this relationship based on age, as has been observed for other infections [10].

## Results

### Population

There were 3851 patients included in the study. 558 (14%) were underweight (BMI < 20), 1310 (34%) were normal weight (BMI 20–25), 1028 (27%) were overweight (BMI 25–30), 539 (14%) had class I obesity (BMI 30–35), 235 (6%) had class II obesity (BMI 35–40), 102 (3%) had class III obesity (BMI 35–40), and 79 (2%) had extreme obesity (BMI > 45) (Table 1). Additional file 2: Table S1.

**Table 1:** Clinical and demographic characteristics of hospitalized patients with CDI patients, stratified by mortality

	No death within 30 days, N (%)	Death within 30 days, N (%)	p value
**BMI**			
< 20	519 (14.9%)	39 (10.5%)	0.32
20–25	1175 (33.8%)	135 (36.2%)	
25–30	920 (26.5%)	108 (29.0%)	
30–35	490 (14.1%)	49 (13.1%)	
35–40	213 (6.1%)	22 (5.9%)	
40–45	92 (2.6%)	10 (2.7%)	
> 45	69 (2.0%)	10 (2.7%)	
**Sex**			
Female	1830 (52.6%)	194 (52.0%)	0.82
Male	1648 (47.4%)	179 (48.0%)	
**Age**			
18–55 years	1102 (31.7%)	56 (15.0%)	< 0.01
56–75 years	1522 (43.8%)	180 (48.3%)	
≥ 76 years	854 (24.6%)	137 (36.7%)	
**Race/ethnicity**			
Black	356 (10.2%)	37 (9.9%)	1.00
Hispanic	385 (11.1%)	41 (11.0%)	
White	1930 (55.5%)	207 (55.5%)	
Other	807 (23.2%)	88 (23.6%)	
**Charlson comorbidity index**			
0–3 points	1439 (41.4%)	62 (16.6%)	< 0.01
4–6 points	1682 (48.4%)	249 (66.8%)	
> 7 points	357 (10.3%)	62 (16.6%)	
**Vital signs**			
Temperature < 35, > 38 °C	578 (24.7%)	98 (41.0%)	< 0.01
Heart rate > 100 beats/min	1728 (56.5%)	247 (77.4%)	< 0.01
MAP < 65 mmHg	940 (30.9%)	193 (62.1%)	< 0.01
**Lab values**			
WBC count > 15 × 10^3^/μL	1100 (31.6%)	208 (55.8%)	< 0.01
Hematocrit < 37.2%	2375 (91.2%)	308 (96.9%)	< 0.01
Platelet count < 156 × 10^3^/μL	1302 (37.4%)	209 (56.0%)	< 0.01
Total bilirubin > 1.3 mg/dL	308 (14.0%)	86 (33.1%)	< 0.01
Albumin < 3 g/dL	1306 (44.4%)	241 (70.5%)	< 0.01
Creatinine > 1.5 mg/dL	1087 (31.4%)	218 (58.4%)	< 0.01
**Hospitalization**			
Vasopressor use	533 (15.3%)	140 (37.5%)	< 0.01
ICU stay	914 (26.3%)	208 (55.8%)	< 0.01

Baseline characteristics of patients with *Clostridioides difficile* infection (n = 3851), stratified based on death within 30 days

CDI, *Clostridioides difficile* infection; BMI, body mass index; MAP, mean arterial pressure; WBC, white blood cell; ICU, intensive care unit

### Baseline characteristics and death

Overall, 373 (9.7%) patients died within 30 days of the index test for CDI. The median BMI was 25.6 (IQR 7.5) in those who died within 30 days of CDI testing versus 25.2 (IQR 8.3) in those who did not die (rank-sum p = 0.19). When BMI was classified into categories, there was no crude association between BMI and death (Table 1, chi-squared p = 0.32). Those who died were older, had more comorbidities, and had higher frequency of vasopressor use and ICU requirement (Table 1). Those who died were also more likely to have abnormal vital signs (temperature, heart rate, mean arterial pressure) and abnormal lab values (leukocytosis, low hematocrit, low platelets, increased total bilirubin, low albumin, and increased serum creatinine).

### Multivariable model for obesity and death

To identify the factors that independently predicted short-term risk for death among hospitalized patients with CDI, we constructed a multivariable model. Included in this model a priori were creatinine, albumin, WBC count, and ICU stay at the time of index CDI testing. In this model, BMI was not associated with risk for death in any BMI category (Table 2). Significant predictors of death in the final model were older age, albumin < 3 g/dL, and increased comorbidities (Table 2). When BMI was reclassified as an ordered variable, there was also no risk for death associated with BMI (Cochran-Armitage p = 0.42). Finally, BMI was reclassified by condensing it into BMI > 30 and BMI > 40. Again, there was no significant association between BMI and 30-day mortality (aOR 0.96, 95% CI 0.69 to 1.34 for BMI > 30 vs BMI 20–30; aOR 1.02, 95% CI 0.53 to 1.87 for BMI > 40 vs BMI 20–30).

**Table 2:** Final multivariable model including BMI and predictors of 30 day mortality among those with CDI

	Adjusted odds ratio	95% confidence interval
**Body mass index (BMI)**		
BMI < 20	0.62	0.35, 1.03
BMI 20–25	Reference	
BMI 25–30	0.87	0.61, 1.24
BMI 30–35	0.83	0.52, 1.30
BMI 35–40	1.04	0.55, 1.87
BMI 40–45	0.94	0.35, 2.20
BMI > 45	0.97	0.38, 2.21
**Age**		
Age 18–55	Reference	
Age 56–75	1.55	1.03, 2.37
Age ≥ 76	2.17	1.36, 3.52
**Comorbidities**		
Charlson comorbidity index 0–3 points	Reference	
Charlson comorbidity index 4–6 points	2.05	1.37, 3.11
Charlson comorbidity index > 7 points	1.97	1.19, 3.29
ICU stay	1.74	1.27, 2.39
**Lab values**		
Creatinine > 1.5 mg/dL	1.88	1.39, 2.56
Albumin < 3 g/dL	2.12	1.58, 2.87
WBC count > 15 × 10^3^/μL	1.77	1.30, 2.42
Platelet count < 156 × 10^3^/μL	1.69	1.24, 2.31
Total bilirubin > 1.3 mg/dL	2.01	1.43, 2.82
**Vital signs**		
Heart rate > 100 beats/min	1.72	1.19, 2.51
MAP < 65 mmHg	1.39	1.00, 1.93

BMI, body mass index; WBC, white blood cell; ICU, intensive care unit; MAP, mean arterial pressure

### Age-stratified analyses

**Fig. 1:**
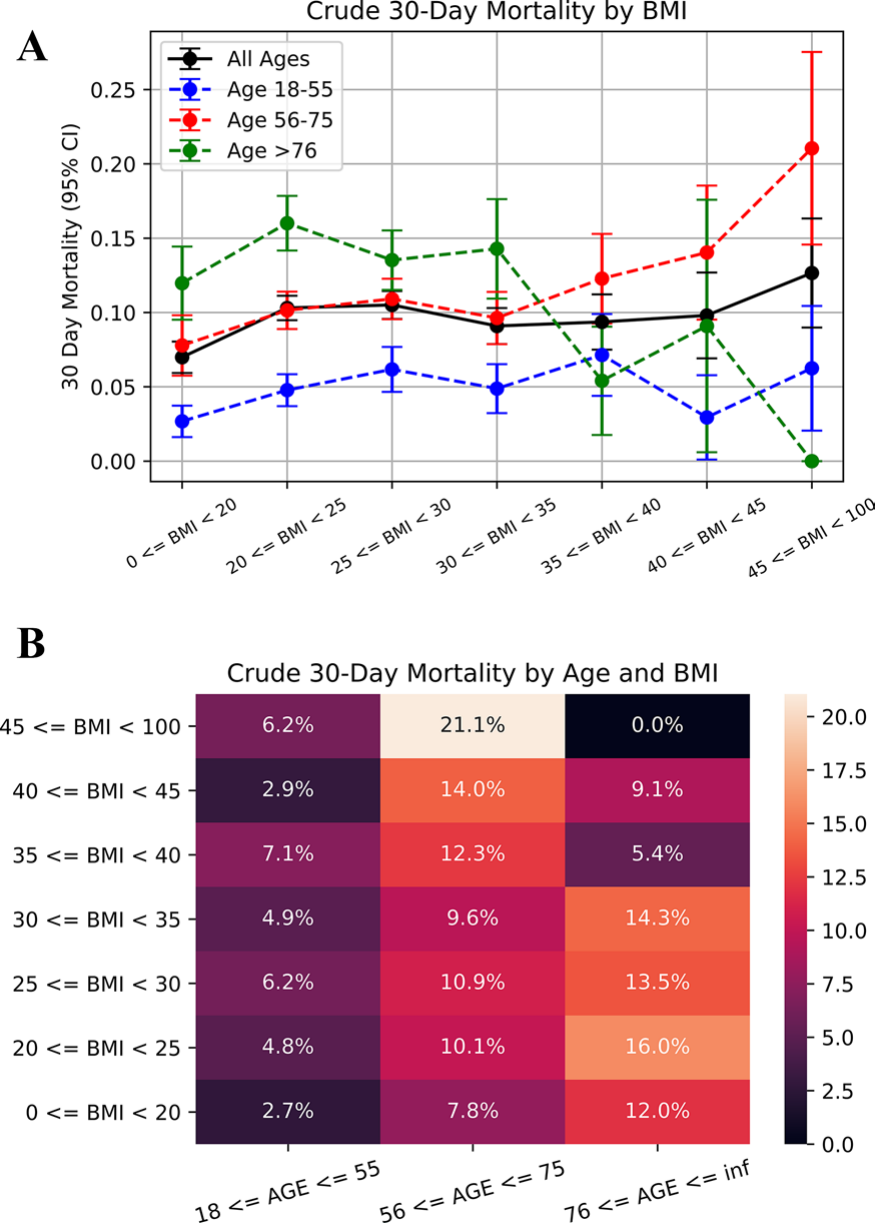
Relationship between BMI and mortality, stratified by age. **A** Trends for mortality based on BMI are visualized, after stratifying by age. Whiskers show 95% confidence intervals. Blue: age group 18–55, red: age group 56–75, green: age group ≥ 76, and black: all ages. **B** An alternative visualization of the BMI-death relationship as a heatmap coded for the crude 30-day mortality rate. Again, data has been stratified by age. Dark colors represent lower mortality rates; light colors represent higher mortality rates. BMI, body mass index; CI, confidence interval

To account for the possibility of an age-based interaction in the BMI-death relationship, we stratified the study population into three age categories: 18 to 55, 56 to 75, and > 75 years old. When interaction terms were entered into the final model for BMI as a categorical variable and for age in its three strata, there was no evidence of interaction (i.e., p = NS for all interaction terms). Nonetheless, we elected to perform stratified analyses to better understand the BMI-death relationship after accounting for age. BMI was again classified into categories (< 20, 20–25, 25–30, 30–35, 35–40, 40–45, and > 45). When the BMI-death relationship was examined visually, there was some evidence of a trend towards increased risk for death among those who were younger and obese and decreased risk for death among those who were old and obese (Fig. 1, Cochran-Armitage p = 0.33 for ≤ 55 years old, 0.14 for age 56–75, 0.37 for age > 75). In an age-stratified analysis, among those 18 to 55 years old, BMI of 35–40 was associated with increased risk for death compared to BMI of 20–25 (aOR 4.58, 95% CI 1.20–16.1). However, there was low confidence in this estimate, and this was the only BMI-death association observed in multiple strata (Table 3). When age was reorganized into five quintiles (18–34, 35–51, 52–68, 69–85, and > 85 years old), these results were substantively unchanged.

**Table 3:** Age-stratified relationship between BMI and 30 day mortality among hospitalized patients with CDI, after adjusting for other factors

	Age strata		
	Age 18–55	Age 56–75	Age > 75
Cochran-armitage p for trend			
BMI (per category increase)^a^	0.33	0.14	0.37
Adjusted odds ratios and 95% confidence intervals			
BMI categories			
BMI < 20	0.89 (0.27–2.63)	0.50 (0.19–1.17)	0.56 (0.23–1.27)
BMI 20–25	Reference	Reference	Reference
BMI 25–30	0.86 (0.31–2.27)	0.85 (0.50–1.43)	0.85 (0.47–1.53)
BMI 30–35	1.12 (0.36–3.19)	0.69 (0.35–1.30)	1.04 (0.44–2.33)
BMI 35–40	4.58 (1.20–16.06)	0.90 (0.38–1.95)	0.23 (0.01–1.30)
BMI 40–45	1.10 (0.06–7.03)	1.04 (0.31–2.96)	0.68 (0.03–5.34)
BMI > 45	1.19 (0.16–5.48)	1.36 (0.41–4.01)	–

Odds ratios adjusted for Charlson comorbidity index, increased serum creatinine, low albumin, increased WBC count, ICU stay, abnormal heart rate, abnormal MAP, abnormal platelet count, and abnormal total bilirubin

BMI, body mass index; WBC, white blood cell; ICU, intensive care unit; MAP, mean arterial pressure

^a^The Cochran-Armitage test was used to assess trend across BMI categories (same categories as specified in table)

### Hospital-free days

To determine whether healthcare utilization varied based on BMI, we examined hospital-free days. There was no significant difference in hospital-free days between BMI groups (Kruskal–Wallis p = 0.92, Table 4).

**Table 4:** Hospital-free days, stratified by BMI category

BMI categories	Hospital-free days Median (interquartile range)
BMI < 20	19.0 (15.24)
BMI 20–25	19.0 (17.22)
BMI 25–30	19.0 (19.14)
BMI 30–35	19.0 (19.11)
BMI 35–40	19.98 (14.36)
BMI 40–45	17.07 (16.87)
BMI > 45	17.0 (24.60)

BMI, body mass index

### Additional analyses

Additional analyses were performed for community-acquired vs healthcare-associated CDI, based on institution, based on ethnicity/race, and based on ICU stay. Among patients with community-acquired CDI (n = 2790), there was no significant association between BMI category and 30-day mortality in any category or when BMI was examined as an ordered variable. Among patients with healthcare-associated CDI (n = 1061), there was a weak association between BMI < 20 and decreased 30-day mortality (aOR 0.36, 95% CI 0.12–0.91). There was also a weak association between increasing BMI and mortality among BWH patients (aOR 1.23 per increase in BMI category, 95% CI 1.04–1.44). Otherwise, there were no significant associations between BMI category and 30-day mortality in the cohorts when stratified based on institution, ethnicity/race, or hospitalization in the ICU; this was true with BMI classified either as a discrete or ordered variable.

## Discussion

In this retrospective multicenter study including nearly 4000 patients hospitalized with *C. difficile* infection, we found no association between BMI and thirty-day mortality. This finding was very robust. It was true when BMI was classified as a discrete variable to account for the possibility of a non-linear relationship, when BMI was classified more simply as > 30 or > 40, and when BMI was treated as an ordered variable. We had hypothesized that there would be an age-related interaction with BMI (i.e., that being young and obese or old and thin would associate with death compared to being normal weight). Although there were subtle trends in this direction, this hypothesis was not supported by the data. In multiple secondary analyses—based on community-acquired versus healthcare-associated CDI, ICU stay, and other factors—the findings were similar. In addition, we found no significant difference in hospital-free days based on BMI. Currently, there is limited ability to predict adverse outcomes among hospitalized patients with CDI. However, based on these results, it is unlikely that BMI will be a useful factor to further risk-stratify patients with CDI. It also seems unlikely that the obesity epidemic explains much of the rise in CDI observed since the 1990s.

Prior studies have produced some contradictory results related to obesity and CDI. Bishara et al. found that obesity was a risk factor for incident healthcare-associated CDI, while Meier et al*.* found that high BMI was associated with decreased risk of postoperative CDI and Punni et al*.* found no difference in BMI between CDI cases and controls [19–21]. Some studies have suggested that obesity is associated with adverse outcomes in CDI. Nathanson et al. found that being underweight (BMI < 19) or morbidly obese (BMI > 40) were both associated with increased risk of mortality in patients with CDI [22]. Nathanson’s study, like ours, found no interaction between age and BMI [22]. Their cohort was comprised of patients presenting to the emergency department with CDI, therefore focusing on community-acquired infections. Their population had lower BMI (33% with BMI < 19.0 kg/m^2^) and older age (32% older than 75) compared to patients in our study [22]. Similar to Nathanson, Tariq et al. evaluated in-hospital mortality for CDI patients but found that obesity, even morbid obesity, was associated with a *decreased* risk of mortality in CDI [23]. In contrast, our study included both community-acquired and hospital-acquired CDI, with a primary outcome of thirty-day all-cause mortality. The variation in population, time frame, and outcome of interest may account for the difference in our results.

In general, obesity and being underweight are associated with increased risk for all-cause mortality [24, 25]. A review of the effect of BMI on infection outcomes has also shown that obese or morbidly obese patients as well as underweight patients have worse outcomes from infectious diseases than normal weight patients [26]. Most of these studies have involved respiratory tract infections [9–11, 27–31], leading to sepsis [32, 33] and bacteremia [34]. CDI differs from other infections in that it rarely is the direct cause of death. Rather, among hospitalized patients, CDI can contribute to an overall decline in functional status, prolonged hospitalization, and eventual death that is often from sepsis caused by other healthcare-associated infections (e.g., bacteremia) [35]. It is difficult to adjudicate cause of death among hospitalized patients with CDI [36].

Our study found that increased age, elevated creatinine, low albumin, leukocytosis, low platelet count, and elevated total bilirubin were associated with increased risk of 30-day mortality in CDI patients. These findings are consistent with prior studies that have identified increased age, leukocytosis, low albumin, and elevated creatinine as risk factors for severe CDI and mortality in CDI patients [18, 37]. The current IDSA/SHEA recommends using leukocytosis and serum creatinine as well as hypotension, shock, ileus, and megacolon to classify CDI infection severity and guide treatment [38]. The American College of Gastroenterology recommends using a slightly different classification scheme: hypoalbuminemia, leukocytosis, ICU stay, hypotension, fever, serum lactate levels, mental status changes, and end organ failure [39]. Increasing the accuracy of these prediction models can help to allocate hospital resources and target new therapies toward those at greatest risk for CDI mortality. Based on our results, BMI is not likely to prove useful in this regard.

This study has some strengths. It was relatively large, included three unaffiliated medical systems, and used multiple secondary analyses to demonstrate that the results are robust regardless of how BMI is operationalized. CDI was rigorously classified using both a positive PCR result and administration of treatment [39]. Limitations include the retrospective nature of the study and the use of 30-day all-cause mortality, which may be less specific than CDI-attributable mortality. Last, BMI was used as a measure of obesity in this study, but markers of central obesity such as waist-hip ratio may better predict death, especially in elderly or non-white patients [40].

## Conclusions

In sum, this large, retrospective study found no association between BMI and 30 day all-cause mortality or hospital-free days among hospitalized patients with CDI. Future studies seeking to risk-stratify hospitalized patients with CDI may wish to test other novel variables (e.g., platelet count or bilirubin levels) to improve prognostication for hospitalized patients with CDI. Rise in obesity is not likely to explain the concurrent rise in CDI.

## Methods

### Setting

This was a retrospective cohort study across three institutions, Columbia University Irving Medical Center (CUIMC), Brigham and Women’s Hospital (BWH), and NYU Langone Health (NYU). The timeframe of the study, January 2010 to June 2018 for CUIMC and NYU and June 2015 to December 2018 for BWH, reflects the period when Cepheid stool polymerase chain reaction (PCR) tests were used to diagnose CDI in all three institutions. The study received approval from the institutional review boards of CUIMC, BWH, and NYU.

### Population

Hospitalized adults 18 or more years old were included in the study if they had a positive *C. difficile* PCR for the toxin B gene performed on an unformed stool specimen and received appropriate anti-CDI treatment within 48 h of the index test (either before or after). If there were multiple positive stool tests for a patient, the first test was selected in order to study unique individuals. Patients were excluded from the study if they did not have BMI measured at the time of hospital admission, or if the BMI was recorded as less than 10 or greater than 100 (Additional file 1: Fig. S1).

### Obesity

Body mass index was used as a measure of obesity because it was available retrospectively within the electronic medical record. We hypothesized based on prior studies of obesity in patients with infections that the relationship between obesity and mortality in patients with CDI would be non-linear. Thus, we divided patients into groups based on BMI (kg/m^2^) as: < 20 (underweight), 20–25 (normal weight), 25–30 (overweight), 30–35 (class I obesity), 35–40 (class II obesity), 40–45 (class III obesity), and > 45 (extreme obesity). BMI of 20–25, considered normal weight, was set as the reference category. We also performed analyses with BMI classified as an ordered but non-linear variable using the Cochran-Armitage trend test and with BMI condensed into fewer categories in order to increase the sample size within the moderate to severe obesity categories; in these analyses BMI > 30 and BMI > 40 were each compared to a reference category of BMI 20–30.

### Primary outcome

The primary outcome was death from any cause within 30 days after the initial CDI test. Because of adjudication of cause of death is notoriously challenging, we did not attempt to distinguish CDI-related from CDI-unrelated death [41].

### Hospital-free days

Hospital-free days were examined as a continuous measure to describe healthcare utilization [42]. Hospital-free days were calculated as the number of days alive spent outside of the hospital during the 30 day period after diagnosis of CDI. For example, if a patient was discharged on day 5 after CDI diagnosis and died on day 25, the patient had 20 hospital-free days. Hospital-free days were compared between BMI categories using a Kruskal–Wallis test (Additional file 2: Table S1).

### Statistical approach

Demographic and clinical characteristics at the time of the index CDI test were gathered from the electronic medical record using automated queries. Age and Charlson comorbidity index were split into tertiles. Laboratory values were defined categorically as normal or abnormal based on the institutional laboratory reference ranges. For serum creatinine and white blood cell count, cutoffs were established based on the Infectious Diseases Society of America (IDSA) and Society for Healthcare Epidemiology of America (SHEA) CDI disease severity criteria (white blood cell count ≥ 15,000 cells/mL and serum creatinine > 1.5 mg/dL) [38]. To build the final multivariable model for death, BMI was entered and additional variables were added stepwise, retaining those in the final model that independently predicted death (p < 0.05). Creatinine, albumin, WBC count, and ICU stay were included a priori as established predictors of mortality in CDI. Statistically significant differences were defined as p < 0.05. All statistical analysis was performed using Python and R.

### Age-stratified analyses

Thirty-day mortality for each BMI category and age tertile were graphed and visualized on a heatmap to assess for an interaction between age and BMI. An age-stratified logistic regression analysis for BMI and death was performed within each age tertile, with BMI classified as a discrete variable (same categories as above). BMI was also classified as an ordered variable, and relationship to thirty-day mortality was assessed with Cochran-Armitage trend testing.

### Additional analyses

Several additional analyses were performed. First, patients were classified as community-acquired (CA-CDI) versus healthcare-associated CDI (HA-CDI) using a cut-off of whether the index test was performed within < 72 h of hospital admission to define community-acquired CDI. The BMI-death relationship was then reassessed independently for CA-CDI and HA-CDI. Stratified analyses were also performed for each institution separately, each race/ethnicity group, and based on whether patients were hospitalized in the ICU at the time of testing for CDI.

## Supplementary Information

**Additional file 1: Figure S1.** Study Population. Patients were selected from Columbia University Irving Medical Center, New York University Langone Health, and Brigham and Women’s if they had a positive *C. difficile* PCR for the toxin B gene performed on an unformed stool specimen and received appropriate anti-CDI treatment within 48 h of the index test. Patients were excluded from the study if they did not have BMI measured at hospital admission. Patients with BMI recorded as less than 10 or greater than 100 were excluded. Abbreviations: CDI = *Clostridioides difficile* infection, BMI = body mass index.

**Additional file 2: Table S1.** Clinical and demographic characteristics of patient by BMI categories.

## Data Availability

The datasets used and/or analyzed during the current study are available from the corresponding author on reasonable request.

## Abbreviations

aOR: Adjusted odds ratio
BMI: Body mass index
BWH: Brigham and Women’s Hospital
*C. difficile*: *Clostridioides difficile*
CA-CDI: Community-acquired *Clostridioides difficile* infection
CDI: *Clostridioides difficile* infection
CI: Confidence interval
CUIMC: Columbia University Irving Medical Center
HA-CDI: Healthcare-associated *Clostridioides difficile* infection
ICU: Intensive care unit
IDSA: Infectious Diseases Society of America
MAP: Mean arterial pressure
NAP-1: North American Pulsed Field-1
NS: Not significant
NYU: New York University Langone Health
PCR: Polymerase chain reaction
SHEA: Society for Healthcare Epidemiology of America
WBC: White blood cell

## References

[1] Lessa FC, Mu Y, Bamberg WM (2015). Burden of *Clostridium difficile* infection in the United States. N Engl J Med.

[2] Dubberke ER, Olsen MA (2012). Burden of *Clostridium difficile* on the healthcare system. Clin Infect Dis.

[3] Warny M, Pepin J, Fang A (2005). Toxin production by an emerging strain of *Clostridium difficile* associated with outbreaks of severe disease in North America and Europe. The Lancet.

[4] Hensgens MP, Goorhuis A, Dekkers OM (2012). Time interval of increased risk for *Clostridium difficile* infection after exposure to antibiotics. J Antimicrob Chemother.

[5] Stevens V, Dumyati G, Fine LS (2011). Cumulative antibiotic exposures over time and the risk of *Clostridium difficile* infection. Clin Infect Dis.

[6] Shaughnessy MK, Amundson WH, Kuskowski MA (2013). Unnecessary antimicrobial use in patients with current or recent *Clostridium difficile* infection. Infect Control Hosp Epidemiol.

[7] DePestel DD, Aronoff DM (2013). Epidemiology of *Clostridium difficile* infection. J Pharm Pract.

[8] WHO (2020). Obesity and overweight fact sheet.

[9] Fezeu L, Julia C, Henegar A (2011). Obesity is associated with higher risk of intensive care unit admission and death in influenza A (H1N1) patients: a systematic review and meta-analysis. Obes Rev.

[10] Anderson MR, Geleris J, Anderson DR (2020). Body mass index and risk for intubation or death in SARS-CoV-2 infection: a retrospective cohort study. Ann Intern Med.

[11] Hajifathalian K, Kumar S, Newberry C (2020). Obesity is associated with worse outcomes in COVID-19: analysis of early data from New York City. Obesity..

[12] Procaccini C, Jirillo E, Matarese G (2012). Leptin as an immunomodulator. Mol Aspects Med.

[13] Madan R, Guo X, Naylor C (2014). Role of leptin-mediated colonic inflammation in defense against *Clostridium difficile* colitis. Infect Immun.

[14] Mykoniatis A, Anton PM, Wlk M (2003). Leptin mediates *Clostridium difficile* toxin A-induced enteritis in mice. Gastroenterology.

[15] Madan R, Petri WA (2015). Role of obesity and adipose tissue-derived cytokine leptin during *Clostridium difficile* infection. Anaerobe.

[16] McDonald LC, Gerding DN, Johnson S (2018). Clinical practice guidelines for *Clostridium difficile* infection in adults and children: 2017 update by the Infectious Diseases Society of America (IDSA) and Society for Healthcare Epidemiology of America (SHEA). Clin Infect Dis.

[17] Debast SB, Bauer MP, Kuijper EJ (2014). European society of clinical microbiology and infectious diseases: update of the treatment guidance document for *Clostridium difficile *infection. Clin Microbiol Infect.

[18] Abou Chakra CN, Pepin J, Sirard S (2014). Risk factors for recurrence, complications and mortality in *Clostridium difficile* infection: a systematic review. PLoS ONE.

[19] Bishara J, Farah R, Mograbi J (2013). Obesity as a risk factor for *Clostridium difficile* infection. Clin Infect Dis.

[20] Meier K, Nordestgaard AT, Eid AI (2019). Obesity as protective against, rather than a risk factor for, postoperative *Clostridium difficile* infection: a nationwide retrospective analysis of 1,426,807 surgical patients. J Trauma Acute Care Surg.

[21] Punni E, Pula JL, Asslo F (2015). Is obesity a risk factor for *Clostridium difficile* infection?. Obes Res Clin Pract.

[22] Nathanson BH, Higgins TL, McGee WT (2017). The dangers of extreme body mass index values in patients with *Clostridium difficile*. Infection.

[23] Tariq R, Abdullah A, Wahab A (2018). Outcomes of *Clostridium difficile* infection in patients with obesity: a nationwide analysis: 208. Am J Gastroenterol.

[24] Flegal KM, Graubard BI, Williamson DF (2005). Excess deaths associated with underweight, overweight, and obesity. JAMA.

[25] Padwal R, Majumdar SR, Leslie WD (2016). Relationship among body fat percentage, body mass index, and all-cause mortality. Ann Intern Med.

[26] Falagas M, Athanasoulia A, Peppas G (2009). Effect of body mass index on the outcome of infections: a systematic review. Obes Rev.

[27] Tejera A, Santolaria F, Diez M-L (2007). Prognosis of community acquired pneumonia (CAP): value of triggering receptor expressed on myeloid cells-1 (TREM-1) and other mediators of the inflammatory response. Cytokine.

[28] Bochicchio GV, Joshi M, Bochicchio K (2004). A time-dependent analysis of intensive care unit pneumonia in trauma patients. J Trauma Acute Care Surg.

[29] Mehr DR, Binder EF, Kruse RL (2001). Predicting mortality in nursing home residents with lower respiratory tract infection: the Missouri LRI study. JAMA.

[30] Mehr DR, Zweig SC, Kruse RL (1998). Mortality from lower respiratory infection in nursing home residents. J Fam Pract.

[31] Hedlund J, Hansson L-O, örtqvist Å (1995). Short-and long-term prognosis for middle-aged and elderly patients hospitalized with community-acquired pneumonia: impact of nutritional and inflammatory factors. Scand J Infect Dis.

[32] El-Solh A, Sikka P, Bozkanat E (2001). Morbid obesity in the medical ICU. Chest.

[33] Kalfarentzos F, Dougenis D, Cristopoulos D (1987). Prognostic criteria in intra-abdominal sepsis. Int Surg.

[34] Huttunen R, Laine J, Lumio J (2007). Obesity and smoking are factors associated with poor prognosis in patients with bacteraemia. BMC Infect Dis.

[35] Prescott HC, Dickson RP, Rogers MA (2015). Hospitalization type and subsequent severe sepsis. Am J Respir Crit Care Med.

[36] Hota SS, Achonu C, Crowcroft NS (2012). Determining mortality rates attributable to *Clostridium difficile* infection. Emerg Infect Dis.

[37] Henrich TJ, Krakower D, Bitton A (2009). Clinical risk factors for severe *Clostridium difficile*–associated disease. Emerg Infect Dis.

[38] McDonald LC, Gerding DN, Johnson S (2018). Clinical practice guidelines for *Clostridium difficile* infection in adults and children: 2017 update by the Infectious Diseases Society of America (IDSA) and Society for Healthcare Epidemiology of America (SHEA). Clin Infect Dis.

[39] Surawicz CM, Brandt LJ, Binion DG (2013). Guidelines for diagnosis, treatment, and prevention of *Clostridium difficile* infections. Am J Gastroenterol.

[40] Visscher T, Seidell J, Molarius A (2001). A comparison of body mass index, waist–hip ratio and waist circumference as predictors of all-cause mortality among the elderly: the Rotterdam study. Int J Obes.

[41] Sehdev AES, Hutchins GM (2001). Problems with proper completion and accuracy of the cause-of-death statement. Arch Intern Med.

[42] Auriemma CL, Taylor SP, Harhay MO (2021). Hospital-free days: a pragmatic and patient-centered outcome for trials among critically and seriously ill patients. Am J Respir Crit Care Med.

