# Supplementary material for: Obesity is not associated with adverse outcomes among hospitalized patients with Clostridioides difficile infection
Source: Gut Pathog. 2022 Jan 29;14:7. doi: 10.1186/s13099-022-00479-z (PMC8799984; doi:10.1186/s13099-022-00479-z)
Supplement: Supplementary file 1 — Additional file 1: Figure S1. Study Population. Patients were selected from Columbia University Irving Medical Center, New York University Langone Health, and Brigham and Women’s if they had a positive C. difficile PCR for the toxin B gene performed on an unformed stool specimen and received appropriate anti-CDI treatment within 48 h of the index test. Patients were excluded from the study if they did not have BMI measured at hospital admission. Patients with BMI recorded as less than 10 or greater than 100 were excluded. Abbreviations: CDI = Clostridioides difficile infection, BMI = body mass index. [file 13099_2022_479_MOESM1_ESM.docx]

**Additional file 1: Figure S1**

4518 patients with positive CDI test and treatment

643 excluded for missing BMI

3875 CDI patients with BMI measurement

24 excluded for BMI values not validated (BMI < 10, >100)

3851 patients in final dataset

**Figure S1: Study Population**

Patients were selected from Columbia University Irving Medical Center, New York University Langone Health, and Brigham and Women’s if they had a positive *C. difficile* PCR for the toxin B gene performed on an unformed stool specimen and received appropriate anti-CDI treatment within 48 hours of the index test. Patients were excluded from the study if they did not have BMI measured at hospital admission. Patients with BMI recorded as less than 10 or greater than 100 were excluded. Abbreviations: CDI = *Clostridioides difficile* infection, BMI = body mass index
