# Supplementary material for: Obesity is not associated with adverse outcomes among hospitalized patients with Clostridioides difficile infection
Source: Gut Pathog. 2022 Jan 29;14:7. doi: 10.1186/s13099-022-00479-z (PMC8799984; doi:10.1186/s13099-022-00479-z)
Supplement: Supplementary file 2 — Additional file 2: Table S1. Clinical and demographic characteristics of patient by BMI categories. [file 13099_2022_479_MOESM2_ESM.docx]

**Additional file 2: Table S1.** Clinical and demographic characteristics of patient by BMI categories

|  | **BMI < 20, N (%)** | **BMI 20-25, N (%)** | **BMI 25-30, N (%)** | **BMI 30-35, N (%)** | **BMI 35-40, N (%)** | **BMI > 40, N (%)** |
| --- | --- | --- | --- | --- | --- | --- |
| **Sex** |  |  |  |  |  |  |
| Female | 331 (59) | 637 (49) | 483 (47) | 301 (56) | 152 (65) | 120 (66) |
| Male | 227 (41) | 673 (51) | 545 (53) | 238 (44) | 83 (35) | 61 (34) |
| **Age** |  |  |  |  |  |  |
| 18-55 years | 224 (40) | 377 (29) | 243 (24) | 164 (30) | 84 (36) | 66 (36) |
| 56-75 years | 167 (30) | 552 (42) | 504 (49) | 270 (50) | 114 (48) | 95 (53) |
| ≥76 years | 167 (30) | 381 (29) | 281 (27) | 105 (20) | 37 (16) | 20 (11) |
| **Race/Ethnicity** |  |  |  |  |  |  |
| Black | 62 (11) | 124 (9) | 93 (9) | 54 (10) | 31 (13) | 29 (16) |
| Hispanic | 63 (11) | 142 (11) | 120 (12) | 65 (12) | 21 (9) | 15 (8) |
| White | 279 (50) | 753 (58) | 576 (56) | 298 (55) | 131 (56) | 100 (55) |
| Other | 154 (28) | 291 (22) | 239 (23) | 122 (23) | 52 (22) | 37 (21) |
| **Charlson comorbidity index** |  |  |  |  |  |  |
| 0-3 points | 254 (46) | 502 (38) | 363 (35) | 213 (39) | 99 (42) | 70 (39) |
| 4-6 points | 252 (45) | 675 (52) | 543 (53) | 267 (50) | 110 (47) | 84 (46) |
| >7 points | 52 (9) | 133 (10) | 122 (12) | 59 (11) | 26 (11) | 27 (15) |
| **Vital Signs** |  |  |  |  |  |  |
| Temperature <35, >38°C | 84 (22) | 231 (27) | 183 (27) | 112 (30) | 37 (23) | 29 (24) |
| Heart rate > 100 beats/min | 283 (58) | 663 (58) | 528 (58) | 294 (62) | 113 (54) | 94 (59) |
| MAP <65 mmHg | 177 (36) | 361 (32) | 312 (35) | 160 (34) | 54 (26) | 69 (43) |
| **Lab Values** |  |  |  |  |  |  |
| WBC count >15x 10^3^/uL | 184 (33) | 429 (33) | 372 (36) | 180 (33) | 76 (32) | 67 (37) |
| Hematocrit <37.2% | 434 (96) | 905 (91) | 701 (91) | 356 (91) | 161 (88) | 126 (94) |
| Platelet count <156 x10^3^/uL | 194 (35) | 509 (39) | 419 (41) | 226 (42) | 97 (41) | 66 (36) |
| Total bilirubin >1.3 mg/dL | 35 (10) | 121 (15) | 113 (17) | 68 (18) | 29 (18) | 28 (23) |
| Albumin <3 g/dL | 240 (51) | 539 (48) | 409 (47) | 199 (43) | 86 (44) | 74 (46) |
| Creatinine >1.5 mg/dL | 153 (28) | 433 (33) | 359 (35) | 191 (35) | 83 (35) | 86 (48) |
| Vasopressor use | 91 (16) | 217 (17) | 184 (18) | 98 (18) | 43 (18) | 40 (22) |
| ICU stay | 157 (28) | 357 (27) | 309 (30) | 175 (32) | 67 (29) | 57 (31) |
| **Outcomes** |  |  |  |  |  |  |
| Mortality at 30 days | 39 (7) | 135 (10) | 108 (11) | 49 (9) | 22 (9) | 20 (11) |
